# Bis{μ-*N*-[(dimethyl­amino)­dimethyl­sil­yl]-2,6-dimethyl­anilido}-κ^2^ *N*:*N*′;κ^2^ *N*′:*N*-dicopper(I)

**DOI:** 10.1107/S1600536810042881

**Published:** 2010-10-30

**Authors:** Juan Chen

**Affiliations:** a Department of Chemistry, Taiyuan Teachers College, Taiyuan 030031, People’s Republic of China

## Abstract

The title compound, [Cu_2_(C_12_H_21_N_2_Si)_2_], is a binuclear Cu^I^ complex. The dimeric mol­ecule has an inversion center located at the mid-point of the Cu—Cu bond [Cu—Cu = 2.7209 (7) Å]. The bidentate ligand behaves in an *N*:*N*′-bridging mode, coordinating the metal atoms. The N—Cu—N unit is close to being linear [176.60 (8)°]. The two N atoms exhibit different affinities for the metal atom. The Cu—N_amino_ bond is longer than the Cu—N_anilido_ bond by 0.079 Å. The core of the mol­ecule, the [Cu—N—Si—N]_2_ eight-membered ring, adopts a chair configuration.

## Related literature

For related copper(I) compounds, see: Chen *et al.* (1992 ▶); James *et al.* (1998 ▶); Noto *et al.* (2003 ▶); Guo *et al.* (2009 ▶). For related organometallic compounds with analogous anilido ligands, see: Schumann *et al.* (2000 ▶); Chen (2008 ▶, 2009 ▶); Yuan *et al.* (2010 ▶).
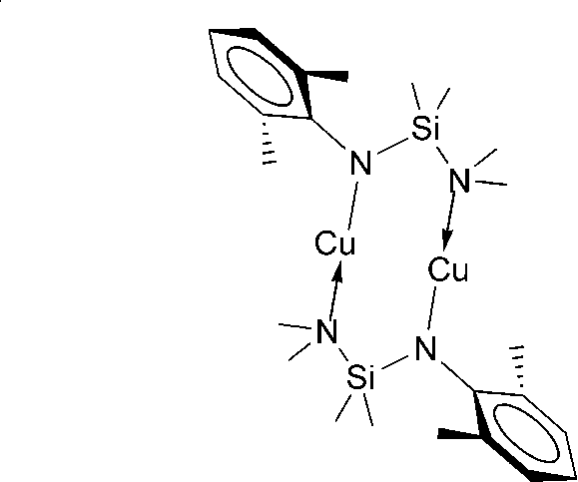


## Experimental

### 

#### Crystal data

[Cu_2_(C_12_H_21_N_2_Si)_2_]
*M* *_r_* = 569.88
Triclinic, 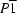

*a* = 8.3609 (18) Å
*b* = 8.4384 (18) Å
*c* = 10.986 (2) Å
α = 94.671 (3)°
β = 97.858 (2)°
γ = 113.824 (2)°
*V* = 694.3 (3) Å^3^
*Z* = 1
Mo *K*α radiation
μ = 1.64 mm^−1^
*T* = 203 K
0.20 × 0.20 × 0.15 mm

#### Data collection

Bruker SMART area-detector diffractometer
Absorption correction: multi-scan (*SADABS*; Sheldrick, 1996 ▶) *T*_min_ = 0.736, *T*_max_ = 0.791
2868 measured reflections
2388 independent reflections
2188 reflections with *I* > 2σ(*I*)
*R*_int_ = 0.013

#### Refinement

*R*[*F* ^2^ > 2σ(*F* ^2^)] = 0.033
*wR*(*F* ^2^) = 0.098
*S* = 1.08
2388 reflections
145 parameters
H-atom parameters constrained
Δρ_max_ = 0.56 e Å^−3^
Δρ_min_ = −0.29 e Å^−3^

### 

Data collection: *SMART* (Bruker, 2000 ▶); cell refinement: *SAINT* (Bruker, 2000 ▶); data reduction: *SAINT*; program(s) used to solve structure: *SHELXS97* (Sheldrick, 2008 ▶); program(s) used to refine structure: *SHELXL97* (Sheldrick, 2008 ▶); molecular graphics: *SHELXTL/PC* (Sheldrick, 2008 ▶); software used to prepare material for publication: *SHELXL97*.

## Supplementary Material

Crystal structure: contains datablocks I, global. DOI: 10.1107/S1600536810042881/vm2052sup1.cif

Structure factors: contains datablocks I. DOI: 10.1107/S1600536810042881/vm2052Isup2.hkl

Additional supplementary materials:  crystallographic information; 3D view; checkCIF report

## Figures and Tables

**Fig. 1.**
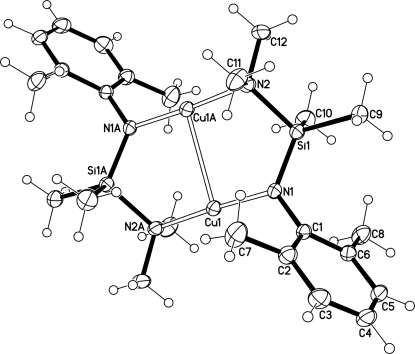
The molecular structure, showing the atom-numbering scheme. Displacement ellipsoids are drawn at the 30% probability level. Symmetry code used to generate second part: 1-x, 2-y, 2-z.

## supplementary crystallographic information

### Comment

In the past decades, considerable attention was paid to monovalent copper amides because of their potential applications in chemical vapor deposition (CVD), organic electroluminescent devices (EL), as well as their structural diversity. The tetranuclear copper(I) amide, [CuN(SiMe_3_)_2_]_4_, has proved to be a useful precursor in these areas (Chen *et al.*, 1992; James *et al.*, 1998; Noto *et al.*, 2003). In contrast to the traditional monodentate amido ligands, the *N*-silylated anilido ligands with a pendant amino group were developed and supposed to be bidentate. They were employed for synthesizing compounds with different metals including Zn (Schumann *et al.*, 2000), Zr (Chen, 2009; Yuan *et al.*, 2010) and Fe (Chen, 2008). Here, the synthesis and crystal structure of a new copper(I) anilido complex will be described.

The molecular structure is illustrated in Fig. 1. The *N*-silylated anilido ligand has an N—Si—N chelating moiety, which is presumed to be a "quasi" conjugated unit owing to *d*–*π* interaction between the Si and N atoms. In the binuclear copper compound, each Cu^I^ atom coordinates to two N from two ligands, one being an anilido group and another being an amino group. Therefore, the bidentate ligand behaves as *N,N'*-µ-bridging mode. Each N—Cu—N unit is close to linear and the two N—Cu—N units are nearly co-planar. The two silyl groups are located above and beneath the plane, respectively, which leads to the "chair" configuration of the [Cu—N—Si—N]_2_ eight-membered ring. The bond lengths N1—Cu1, N2—Cu1A (Cu1A is generated by symmetry operation 1-*x*, 2-*y*, 2-*z*), N1—Si1 and N2—Si1 are 1.848 (2), 1.927 (2), 1.687 (2) and 1.819 (2) Å, respectively. The central Cu—Cu bond is 2.7209 (7) Å, which is comparable to the metal-metal interaction in another reported copper(I) compound (Guo *et al.*, 2009). It is noteworthy that the packing is stablized by a C—H···π interaction between H12A and the phenyl ring C1-C6.

### Experimental

CuCl (0.25 g, 2.50 mmol) was added into the solution of [LiN(SiMe_2_NMe_2_)(2,6-Me_2_C_6_H_3_)]_2_ (0.57 g, 1.25 mmol) in tetrahydrofuran (30 ml) at 273 K. The reaction mixture was warmed to room temperature and kept stirring for 12 h. It was dried in vacuum to remove all volatiles and the residue was extracted with CH_2_Cl_2_ (30 ml). Concentration of the filtrate under reduced pressure and recrystallization in hexane gave the title compound as colorless crystals (yield 0.51 g, 71%).

### Refinement

The methyl H atoms were constrained to an ideal geometry, with C—H distances of 0.97 Å and *U*_iso_(H) = 1.5*U*_eq_(C), but each group was allowed to rotate freely about its C–C, C–N or C–Si bonds. The other H atoms were placed in geometrically idealized positions and constrained to ride on their parent atoms, with C—H distances of 0.94 Å and *U*_iso_(H) = 1.2*U*_eq_(C).

### Crystal data

**Table:**

[Cu_2_(C_12_H_21_N_2_Si)_2_]	*Z* = 1
*M**_r_* = 569.88	*F*(000) = 300
Triclinic, *P*1	*D*_x_ = 1.363 Mg m^−^^3^
Hall symbol: -P 1	Mo *K*α radiation, λ = 0.71073 Å
*a* = 8.3609 (18) Å	Cell parameters from 2538 reflections
*b* = 8.4384 (18) Å	θ = 2.7–27.3°
*c* = 10.986 (2) Å	µ = 1.64 mm^−^^1^
α = 94.671 (3)°	*T* = 203 K
β = 97.858 (2)°	Block, colorless
γ = 113.824 (2)°	0.20 × 0.20 × 0.15 mm
*V* = 694.3 (3) Å^3^	

### Data collection

**Table:**

Bruker SMART area-detector diffractometer	2388 independent reflections
Radiation source: fine-focus sealed tube	2188 reflections with *I* > 2σ(*I*)
graphite	*R*_int_ = 0.013
φ and ω scans	θ_max_ = 25.0°, θ_min_ = 1.9°
Absorption correction: multi-scan (*SADABS*; Sheldrick, 1996)	*h* = −9→9
*T*_min_ = 0.736, *T*_max_ = 0.791	*k* = −10→7
2868 measured reflections	*l* = −12→13

### Refinement

**Table:**

Refinement on *F*^2^	Primary atom site location: structure-invariant direct methods
Least-squares matrix: full	Secondary atom site location: difference Fourier map
*R*[*F*^2^ > 2σ(*F*^2^)] = 0.033	Hydrogen site location: inferred from neighbouring sites
*wR*(*F*^2^) = 0.098	H-atom parameters constrained
*S* = 1.08	*w* = 1/[σ^2^(*F*_o_^2^) + (0.0678*P*)^2^ + 0.0685*P*] where *P* = (*F*_o_^2^ + 2*F*_c_^2^)/3
2388 reflections	(Δ/σ)_max_ = 0.002
145 parameters	Δρ_max_ = 0.56 e Å^−^^3^
0 restraints	Δρ_min_ = −0.29 e Å^−^^3^

### Special details

**Table:**

Geometry. All e.s.d.'s (except the e.s.d. in the dihedral angle between two l.s. planes) are estimated using the full covariance matrix. The cell e.s.d.'s are taken into account individually in the estimation of e.s.d.'s in distances, angles and torsion angles; correlations between e.s.d.'s in cell parameters are only used when they are defined by crystal symmetry. An approximate (isotropic) treatment of cell e.s.d.'s is used for estimating e.s.d.'s involving l.s. planes.
Refinement. Refinement of *F*^2^ against ALL reflections. The weighted *R*-factor *wR* and goodness of fit *S* are based on *F*^2^, conventional *R*-factors *R* are based on *F*, with *F* set to zero for negative *F*^2^. The threshold expression of *F*^2^ > σ(*F*^2^) is used only for calculating *R*-factors(gt) *etc*. and is not relevant to the choice of reflections for refinement. *R*-factors based on *F*^2^ are statistically about twice as large as those based on *F*, and *R*- factors based on ALL data will be even larger.

### Fractional atomic coordinates and isotropic or equivalent isotropic displacement parameters (Å^2^)

**Table:**

	*x*	*y*	*z*	*U*_iso_*/*U*_eq_	
Cu1	0.64341 (4)	0.97861 (4)	0.97011 (2)	0.02987 (15)	
Si1	0.28617 (9)	0.74330 (9)	0.81632 (6)	0.02923 (19)	
N1	0.5057 (3)	0.8549 (3)	0.81832 (18)	0.0271 (4)	
N2	0.2045 (3)	0.9001 (3)	0.87246 (19)	0.0327 (5)	
C1	0.5948 (3)	0.8365 (3)	0.7208 (2)	0.0281 (5)	
C2	0.6808 (3)	0.9786 (3)	0.6594 (2)	0.0358 (6)	
C3	0.7659 (4)	0.9570 (4)	0.5639 (3)	0.0441 (7)	
H3A	0.8229	1.0531	0.5237	0.053*	
C4	0.7696 (4)	0.7998 (4)	0.5261 (3)	0.0464 (7)	
H4A	0.8259	0.7873	0.4599	0.056*	
C5	0.6887 (3)	0.6602 (4)	0.5873 (2)	0.0406 (6)	
H5A	0.6900	0.5517	0.5620	0.049*	
C6	0.6057 (3)	0.6769 (3)	0.6855 (2)	0.0331 (6)	
C7	0.6796 (5)	1.1541 (4)	0.6940 (3)	0.0546 (8)	
H7A	0.7451	1.2353	0.6420	0.082*	
H7B	0.7351	1.1985	0.7805	0.082*	
H7C	0.5578	1.1418	0.6817	0.082*	
C8	0.5327 (4)	0.5259 (4)	0.7550 (3)	0.0454 (7)	
H8A	0.5513	0.4278	0.7179	0.068*	
H8B	0.4064	0.4918	0.7511	0.068*	
H8C	0.5933	0.5601	0.8411	0.068*	
C9	0.1590 (4)	0.6366 (4)	0.6565 (3)	0.0418 (6)	
H9A	0.1911	0.7215	0.5996	0.063*	
H9B	0.0325	0.5928	0.6568	0.063*	
H9C	0.1874	0.5400	0.6302	0.063*	
C10	0.2242 (4)	0.5790 (4)	0.9263 (3)	0.0483 (7)	
H10A	0.2902	0.6345	1.0094	0.072*	
H10B	0.2527	0.4825	0.9000	0.072*	
H10C	0.0977	0.5351	0.9265	0.072*	
C11	0.2158 (4)	1.0308 (4)	0.7866 (3)	0.0482 (7)	
H11A	0.1395	0.9712	0.7070	0.072*	
H11B	0.3377	1.0905	0.7750	0.072*	
H11C	0.1774	1.1156	0.8220	0.072*	
C12	0.0142 (4)	0.8117 (4)	0.8889 (3)	0.0474 (7)	
H12A	−0.0611	0.7507	0.8093	0.071*	
H12B	−0.0229	0.8990	0.9216	0.071*	
H12C	0.0039	0.7283	0.9465	0.071*	

### Atomic displacement parameters (Å^2^)

**Table:**

	*U*^11^	*U*^22^	*U*^33^	*U*^12^	*U*^13^	*U*^23^
Cu1	0.0302 (2)	0.0337 (2)	0.0257 (2)	0.01420 (15)	0.00558 (13)	−0.00019 (14)
Si1	0.0296 (4)	0.0309 (4)	0.0262 (4)	0.0117 (3)	0.0069 (3)	0.0016 (3)
N1	0.0290 (11)	0.0274 (10)	0.0243 (10)	0.0115 (8)	0.0061 (8)	0.0001 (8)
N2	0.0296 (11)	0.0410 (12)	0.0290 (11)	0.0171 (9)	0.0064 (8)	0.0004 (9)
C1	0.0274 (12)	0.0343 (13)	0.0225 (12)	0.0139 (10)	0.0036 (9)	−0.0003 (10)
C2	0.0372 (14)	0.0367 (14)	0.0357 (14)	0.0161 (11)	0.0108 (11)	0.0066 (11)
C3	0.0428 (16)	0.0532 (17)	0.0350 (15)	0.0151 (13)	0.0160 (12)	0.0127 (13)
C4	0.0384 (15)	0.068 (2)	0.0342 (15)	0.0234 (14)	0.0123 (12)	−0.0031 (13)
C5	0.0352 (14)	0.0488 (16)	0.0396 (15)	0.0238 (12)	0.0027 (12)	−0.0089 (12)
C6	0.0297 (13)	0.0367 (14)	0.0334 (14)	0.0171 (11)	0.0012 (10)	−0.0018 (11)
C7	0.073 (2)	0.0350 (16)	0.062 (2)	0.0207 (15)	0.0328 (17)	0.0155 (14)
C8	0.0543 (17)	0.0379 (15)	0.0546 (18)	0.0281 (13)	0.0155 (14)	0.0083 (13)
C9	0.0386 (15)	0.0474 (17)	0.0363 (15)	0.0185 (13)	0.0031 (11)	−0.0070 (12)
C10	0.0548 (18)	0.0427 (16)	0.0496 (18)	0.0171 (14)	0.0232 (14)	0.0150 (13)
C11	0.0650 (19)	0.0586 (19)	0.0360 (15)	0.0418 (16)	0.0069 (13)	0.0073 (13)
C12	0.0282 (14)	0.062 (2)	0.0483 (17)	0.0184 (13)	0.0089 (12)	−0.0085 (14)

### Geometric parameters (Å, °)

**Table:**

Cu1—N1	1.848 (2)	C5—H5A	0.9400
Cu1—N2^i^	1.927 (2)	C6—C8	1.496 (4)
Cu1—Cu1^i^	2.7209 (7)	C7—H7A	0.9700
Si1—N1	1.687 (2)	C7—H7B	0.9700
Si1—N2	1.819 (2)	C7—H7C	0.9700
Si1—C9	1.866 (3)	C8—H8A	0.9700
Si1—C10	1.875 (3)	C8—H8B	0.9700
N1—C1	1.418 (3)	C8—H8C	0.9700
N2—C11	1.491 (4)	C9—H9A	0.9700
N2—C12	1.504 (3)	C9—H9B	0.9700
N2—Cu1^i^	1.927 (2)	C9—H9C	0.9700
C1—C2	1.407 (3)	C10—H10A	0.9700
C1—C6	1.413 (3)	C10—H10B	0.9700
C2—C3	1.385 (4)	C10—H10C	0.9700
C2—C7	1.503 (4)	C11—H11A	0.9700
C3—C4	1.371 (4)	C11—H11B	0.9700
C3—H3A	0.9400	C11—H11C	0.9700
C4—C5	1.381 (4)	C12—H12A	0.9700
C4—H4A	0.9400	C12—H12B	0.9700
C5—C6	1.387 (4)	C12—H12C	0.9700
			
N1—Cu1—N2^i^	176.60 (8)	C2—C7—H7A	109.5
N1—Cu1—Cu1^i^	88.78 (6)	C2—C7—H7B	109.5
N2^i^—Cu1—Cu1^i^	94.26 (6)	H7A—C7—H7B	109.5
N1—Si1—N2	107.39 (11)	C2—C7—H7C	109.5
N1—Si1—C9	112.14 (12)	H7A—C7—H7C	109.5
N2—Si1—C9	108.35 (12)	H7B—C7—H7C	109.5
N1—Si1—C10	116.21 (12)	C6—C8—H8A	109.5
N2—Si1—C10	102.61 (12)	C6—C8—H8B	109.5
C9—Si1—C10	109.44 (14)	H8A—C8—H8B	109.5
C1—N1—Si1	125.19 (16)	C6—C8—H8C	109.5
C1—N1—Cu1	117.77 (15)	H8A—C8—H8C	109.5
Si1—N1—Cu1	116.05 (11)	H8B—C8—H8C	109.5
C11—N2—C12	107.8 (2)	Si1—C9—H9A	109.5
C11—N2—Si1	112.03 (17)	Si1—C9—H9B	109.5
C12—N2—Si1	112.00 (17)	H9A—C9—H9B	109.5
C11—N2—Cu1^i^	108.72 (18)	Si1—C9—H9C	109.5
C12—N2—Cu1^i^	110.13 (16)	H9A—C9—H9C	109.5
Si1—N2—Cu1^i^	106.11 (10)	H9B—C9—H9C	109.5
C2—C1—C6	117.9 (2)	Si1—C10—H10A	109.5
C2—C1—N1	120.9 (2)	Si1—C10—H10B	109.5
C6—C1—N1	121.1 (2)	H10A—C10—H10B	109.5
C3—C2—C1	119.9 (2)	Si1—C10—H10C	109.5
C3—C2—C7	119.0 (2)	H10A—C10—H10C	109.5
C1—C2—C7	121.2 (2)	H10B—C10—H10C	109.5
C4—C3—C2	122.1 (3)	N2—C11—H11A	109.5
C4—C3—H3A	119.0	N2—C11—H11B	109.5
C2—C3—H3A	119.0	H11A—C11—H11B	109.5
C3—C4—C5	118.6 (2)	N2—C11—H11C	109.5
C3—C4—H4A	120.7	H11A—C11—H11C	109.5
C5—C4—H4A	120.7	H11B—C11—H11C	109.5
C4—C5—C6	121.3 (3)	N2—C12—H12A	109.5
C4—C5—H5A	119.3	N2—C12—H12B	109.5
C6—C5—H5A	119.3	H12A—C12—H12B	109.5
C5—C6—C1	120.1 (2)	N2—C12—H12C	109.5
C5—C6—C8	119.1 (2)	H12A—C12—H12C	109.5
C1—C6—C8	120.7 (2)	H12B—C12—H12C	109.5
			
N2—Si1—N1—C1	−134.55 (18)	Si1—N1—C1—C2	118.2 (2)
C9—Si1—N1—C1	−15.6 (2)	Cu1—N1—C1—C2	−73.7 (3)
C10—Si1—N1—C1	111.3 (2)	Si1—N1—C1—C6	−64.2 (3)
N2—Si1—N1—Cu1	57.14 (15)	Cu1—N1—C1—C6	103.9 (2)
C9—Si1—N1—Cu1	176.06 (11)	C6—C1—C2—C3	2.8 (4)
C10—Si1—N1—Cu1	−57.01 (17)	N1—C1—C2—C3	−179.5 (2)
N2^i^—Cu1—N1—C1	−45.8 (14)	C6—C1—C2—C7	−178.3 (2)
Cu1^i^—Cu1—N1—C1	160.73 (16)	N1—C1—C2—C7	−0.7 (4)
N2^i^—Cu1—N1—Si1	123.5 (13)	C1—C2—C3—C4	0.0 (4)
Cu1^i^—Cu1—N1—Si1	−30.06 (11)	C7—C2—C3—C4	−178.9 (3)
N1—Si1—N2—C11	65.4 (2)	C2—C3—C4—C5	−1.3 (4)
C9—Si1—N2—C11	−55.9 (2)	C3—C4—C5—C6	−0.3 (4)
C10—Si1—N2—C11	−171.6 (2)	C4—C5—C6—C1	3.1 (4)
N1—Si1—N2—C12	−173.29 (16)	C4—C5—C6—C8	−175.2 (2)
C9—Si1—N2—C12	65.4 (2)	C2—C1—C6—C5	−4.3 (3)
C10—Si1—N2—C12	−50.3 (2)	N1—C1—C6—C5	178.0 (2)
N1—Si1—N2—Cu1^i^	−53.09 (14)	C2—C1—C6—C8	174.0 (2)
C9—Si1—N2—Cu1^i^	−174.41 (11)	N1—C1—C6—C8	−3.6 (4)
C10—Si1—N2—Cu1^i^	69.89 (14)		

Symmetry codes: (i) −*x*+1, −*y*+2, −*z*+2.
